# The C-Type Lectin Domain Gene Family in *Aedes aegypti* and Their Role in Arbovirus Infection

**DOI:** 10.3390/v10070367

**Published:** 2018-07-12

**Authors:** Zach N. Adelman, Kevin M. Myles

**Affiliations:** Department of Entomology and Agrilife Research, Texas A&M University, College Station, TX 77843, USA; mylesk@tamu.edu

**Keywords:** *Aedes aegypti*, arbovirus, C-type lectin, dengue, mosquito, immunity, host factor

## Abstract

Several medically important flaviviruses that are transmitted by mosquitoes have been shown to bind to the C-type lectin fold that is present in either vertebrate or invertebrate proteins. While in some cases this interaction is part of a neutralizing anti-viral immune response, many reports have implicated this as critical for successful virus entry. Despite the establishment of mosquito C-type lectin domain containing proteins (CTLDcps) as known host factors in assisting the infectious process for flaviviruses, little is known about the structural characteristics of these proteins and their relationships to each other. In this report, we describe the manual annotation and structural characterization of 52 *Aedes aegypti* CTLDcps. Using existing RNAseq data, we establish that these genes can be subdivided into two classes: those highly conserved with expression primarily in development (embryo/early larvae) and those with no clear orthologs with expression primarily in late larvae/pupae or adults. The latter group contained all CTLDcps that are regulated by the Toll/Imd immune pathways, all known microbiome-regulating CTLDcps, and almost all CTLDcps that are implicated as flavivirus host factors in *A. aegypti*. Finally, we attempt to synthesize results from multiple conflicting gene expression profiling experiments in terms of how flavivirus infection changes steady-state levels of mRNA encoding CTLDcps.

## 1. Introduction

Medically important flaviviruses, such as dengue virus (DENV), Zika virus (ZIKV), West Nile encephalitis virus (WNV), Japanese encephalitis virus (JEV), and yellow fever virus (YFV) are transmitted to humans through the bite of an infected mosquito vector. *A. aegypti* is the primary vector of DENV, ZIKV, and YFV, while mosquitoes of the genus Culex, such as *C. quinquefasciatus*, vector WNV, and JEV. In all cases, these arthropod-borne viruses (arboviruses) must escape the hostile environment of the mosquito gut and complete multiple rounds of replication in mosquito cells of the gut and body before a sufficient number of virus particles reach the salivary ducts where they can be passed on to a new vertebrate host. Intriguingly, a family of proteins containing a C-type lectin domain (CTLD) has been implicated in binding to the glycosylated surface of flaviviruses, such as WNV, DENV, and JEV and promoting their entry (and thus replication) into mosquito cells [1,2,3]. While casually referred to as C-type lectins, proteins that can bind certain sugars in the presence of calcium, the superfamily of C-type lectin domain containing proteins (CTLDcps) is far more broad, with many members that may bind small molecules other than sugars or mediate protein-protein interactions (reviewed in [4]).

With the publication of the first draft of the *A. aegypti* genome in 2007, 39 proteins were identified as containing a CTLD [5]. Comparison of these proteins with both Drosophila and *A. gambiae* CTLDcps revealed a small number of orthologous trios (*n* = 9), as well as multiple species-specific expansions in flies and mosquitoes. Each CTLDcg was named with a common root (CTL), a potential extension (MA or GA if residues indicated a likelihood for binding mannose or galactose, respectively), and a number [5]. Phylogenetic position or the presence or other conserved domains and overall protein structure was not considered. Shortly thereafter, Cheng et al. [1] performed a targeted RNAi screen to identify those CTLDcps that were involved in regulating WNV infection of *A. aegypti* and *C. quinquefasciatus* mosquitoes. Similar targeted screens were reported for genes that are involved in DENV replication [3], JEV replication [2], and those that effect the microbiome [6]. These reports provide alternative names for 34 *A. aegypti* genes encoding CTLDcps based on the convention “mosGCTL” and a number; where “mos” indicates “mosquito”; and, G indicated galactose-binding and CTL is as above. Several difficulties arise from this convention, in addition to those that are raised above. The generic term “mosquito” implies that orthology can be established across all mosquito species, where this is not the case [5], while CTLDcps are not likely to be all galactose-binding (indeed, some are predicted mannose-binding and others are likely to be incapable of binding any sugars). Finally, two novel CTLDcps were characterized in terms of their ability to in inhibit the melanization immune response in *A. aegypti* [7]. Both of the proteins were found to be fusions of a C-terminal CTLD with an N-terminal serine protease domain and were named CLSP1 and CLSP2, accordingly. In this case, the names chosen do denote structural information (although the domain order is reversed in the name). Combined with changes in the gene ID annotation over time as part of shifting from genome assembly version AaegL1 to AaegL5 from 2007 to 2018, and one can see quite quickly how difficult it is at present to synthesize information across different reports that all use different names. For example, CLSP2 was published as gene AAEL011616 (identical to AAEL014385), but is now AAEL019633, and is also mosGCTL-29/mosGCTL-33 and CTLGA9.

As an alternative to these naming schemas, Rao et al. [8] developed a structure-based convention for describing CTLDcps from the lepidopterans *Manduca sexta* [8] and *Bombyx mori* [9]. In this convention, CTLDcps would be classified as CTLD-S (one CTLD domain and no other domain), IML (immunolectin with two CTLD domains), and CTLD-X (one CTLD and one or more additional unrelated domains). Rao et al. [8] recommend avoiding the designation CTL (as well as any MA or GA designations), as the sugar-binding properties of most CTLDcps are unknown. Here, we re-evaluate the CTLDcps of *A. aegypti* and report on the structure, phylogenetic placement, and mRNA expression of 52 CTLD encoding genes. CTLDcps were classified into structure-based groups, as described by Rao et al. [8], with 34 members of CTLD-S, and six members of CTLD-X. Additionally, we distinguish three genes as members of the new group CTLD-SP and nine members of a new group CTLD-E. Members of CTLD-X and CTLD-E are strongly conserved amongst mosquitoes and other insects, and they are largely developmentally expressed. In contrast, members of CTLD-S and CTLD-SP are subject to species-specific expansions and they are largely expressed from late larval through adult stages. We discuss these new observations in the context of prior work on the role of CTLDcps and arbovirus infection of mosquitoes.

## 2. Materials and Methods

### 2.1. C-Type Lectin Identification

Predicted proteins containing the C-type lectin domain (CTLD; cd00037) were downloaded from Vectorbase [10] for *A. aegypti* (AaegL5.1), *A. albopictus* (AaloF1.2), *C. quinquefasciatus* (CpipJ2.4), and *A. gambiae* (AgamP4.9). To identify any additional CTLDcps not yet annotated in the *A. aegypti* genome, the CTLD region was identified from all predicted CTLD proteins using the batch function of the NCBI CD-Search tool [11]. Coordinates for each CTLD were used to extract the corresponding fasta sequence using the getfasta utility in BedTools (v2.25.0) [12]. The resulting multi-fasta file was used as a query to search the *A. aegypti* chromosomal assembly (AaegL5) using the tblastn function of BLAST+ (v2.2.26), with an evalue cutoff of 1 × 10^−5^. The resulting blast output was sorted based on chromosomal position and overlapping intervals were combined using bedtools merge. Each interval was inspected manually using the Webapollo [13] function implemented through Vectorbase, with gene models modified or added as needed based on existing RNAseq data available in Vectorbase. Where CTLDcps were identified but had no corresponding gene model in the AaegL5.1 annotation, if a corresponding protein sequence was found in a deprecated gene annotation set (AaegL3.3 or AaegL1.2) the previous Vectorbase ID was re-established.

### 2.2. Structural Annotation of A. aegypti CTLD Encoding Genes

The final list of manually curated protein predictions was resubmitted to the NCBI CD-Search tool to identify all known conserved domains. Signal peptides and transmembrane regions were predicted using the Phobius webserver [14] and/or SignalP [15]. Coordinates for predicted conserved domains, signal peptides, and transmembrane domains were drawn using a local implementation of the Illustrator for Biological Sequences (Version 1.0) [16].

### 2.3. Phylogenetic Analysis

CTLD domains from manually curated *A. aegypti* predicted proteins and Vectorbase gene models for *A. albopictus*, *C. quinquefasciatus,* and *A. gambiae* were extracted, as above, except that up to five amino acids upstream and downstream of the predicted CTLD domain were included as well (this was not possible in all cases, due to some N- or C-terminal truncations, or simply the C-terminus of the protein occurring within 5 a.a. of the CTLD). CTLD domains were aligned using Muscle (−2.9 gap open; −0.2 gap extend penalties) in MEGA6.0 [17]. The resulting alignment was used to build a tree using the Neighbor-Joining method as implemented in MEGA6.0 using the pairwise deletion option and 2000 bootstrap replicates.

### 2.4. Gene Expression Analysis

To obtain developmental transcriptome data for all CTLDcgs, all 42 mRNAseq libraries first described by Akbari et al. [18] and subsequently remapped to the AaegL5 assembly were downloaded from the Vectorbase FTP server. Raw counts per gene per mapped library were obtained using featureCounts [19]; raw counts were normalized by transcript length and library size (linear normalization) to obtain Fragments per kilobase per million (FPKM) values. FPKM values were log10-transformed [0 values were left as zero, values greater than 0 were treated as log10 of (1 + FPKM)] to keep all the values above zero and to avoid contradictions. Log10-transformed values were analyzed via hierarchical clustering using Morpheus (https://software.broadinstitute.org/morpheus). Additionally, gene identifiers that were associated with CTLD proteins from prior (AaegL3.3 or AaegL1.2) and current (AaegL5.1) annotations were combined in a single non-redundant list and used to identify the corresponding gene expression values from other published microarray [20,21,22,23] and RNAseq [24,25,26] experiments.

## 3. Results

### 3.1. Identification and Classification of A. aegypti CTLDcps

To identify all proteins containing a CTLD in the recently resequenced chromosomal assembly of *A. aegypti* (AaegL5), we used as query the CTLD domains of the 39 previously described CTLs, as described by Waterhouse et al. [5]. After merging overlapping intervals and examining each target region, we confirm the gene models for 52 CTLDcps. Of these, 38 retained a common Vectorbase gene identifier with earlier annotations, eight have new gene identifiers, and six are new genes. Seven genes encoding CTLDcps that were present in prior annotations and were omitted from the AaegL5.1 geneset were restored. Manual curation through Webapollo confirmed that all are in fact present in the genome and expressed. We identified one instance of a fragmented gene, as both RNAseq data and phylogenetic comparisons [8] supported the merger of genes AAEL023473/AAEL019448 (previously AAEL011402/AAEL011403). A complete list of all 52 CTLDcps, along with their genome location, predicted protein length and prior gene identifiers/names is presented in Table S1.

**Figure 1:**
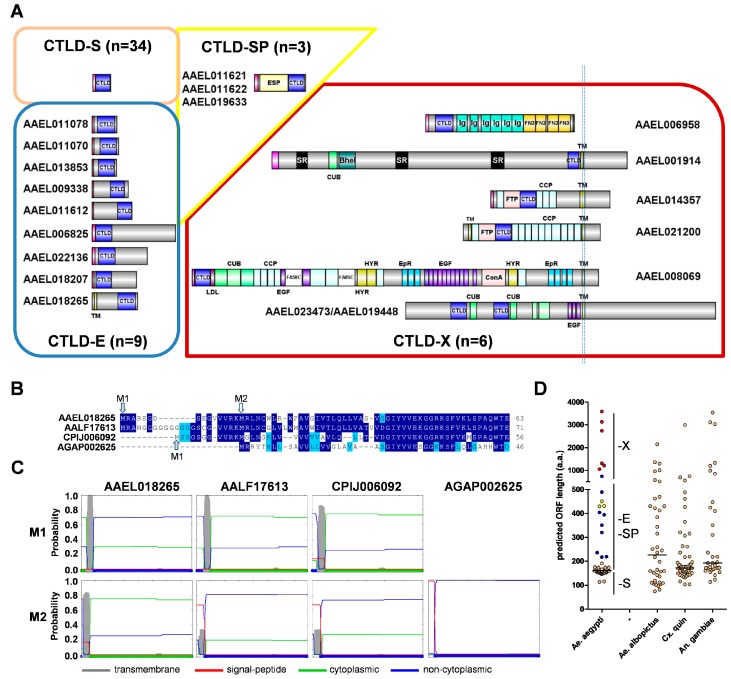
*A. aegypti* C-type lectin domain containing proteins (CTLDcps). (**A**) Primary structure and categorization of CTLDcps. CTLD-S (orange box), CTLD-E (blue box), CTLD-SP (yellow box), and CTLD-X (red box) are indicated. All of the models were drawn to scale using IBS v1.0.3. Predicted signal peptides are indicated at the N-terminus of each sequence (pink), along with predicted transmembrane spanning domains (TM; gold), CTLD (dark blue), and elastase-serine protease (ESP). For CTLD-X members, domains are as indicated and the reader is directed to supplemental Table S1 for a full list of domains including accession numbers for each; (**B**) Alignment of the N-terminus of CTLD-E gene AAEL018265 and its orthologs from other mosquitoes. Identical (dark blue) and similar (light blue) residues are indicated. Initiation methionine (M1) and alternative methionine (M2) are indicated; (**C**) Signal Peptide/transmembrane domain prediction output from Phobius [14] for CTLD-E gene AAEL018265 and its orthologs based on the M1 or M2 methionines; and, (**D**) Predicted amino acid lengths of CTLD genes in *A. aegypti* and three other mosquitoes, by category. For *A. aegypti*, CTLD-S (orange), CTLD-E (blue), CTLD-SP (yellow), and CTLD-X (red) groups are indicated. Horizontal line indicates the median length.

Next, we sought to categorize each CTLDcp based on its predicted size and domain structure (Figure 1A). Thirty-four CTLDcps consisted of a signal peptide and a CTLD domain; these were considered to be members of the CTLD-S group, as described by Rao et al. [8]. Nine additional CTLDcps with no other identifiable domains, but with N-terminal or C-terminal extensions ranging from 31 to 684 amino acids, were placed into a new group, CTLD-E (Figure 1A). This differs from Rao et al. [8], who observed similar extended regions in CTLDcps, but considered these as CTLD-S members as well. However, the mRNA expression profiles and phylogenetic position of these proteins, as discussed below, support their consideration as a separate subgroup. In essence, they are CTLDcps with additional domains, but the function of these linked domains has not yet been deciphered. Of these nine CTLD-E genes, six have primarily N-terminal extensions, while three have C-terminal extensions relative to the CTLD. All CTLD-S proteins and eight of nine CTLD-E proteins are predicted to enter the secretory pathway, while just one (AAEL018265) was predicted to be membrane associated. We sought additional evidence concerning this prediction, as during the annotation process we identified several cases where the use of a secondary methionine for initiation (perhaps in a better context) resulted in a proper signal peptide prediction that was otherwise masked. Following the identification of orthologous genes from other mosquitoes (see Section 3.3 below), we aligned the CTLD-E protein encoded by gene AAEL018265 with its 1:1 orthologs and examined the N-terminal region (Figure 1B). For the three culicine mosquitoes, two methionines were identified within the first 12–22 residues, while the *A. gambiae* ortholog contained only a single methionine. Manual inspection of the upstream genome region failed to reveal any additional methionines, indicating that the Anopheles gene model is correct as is. While the *A. gambiae* ortholog was predicted to enter the secretory pathway with high confidence, all three culicine orthologs are predicted instead to be membrane associated when initiating at the first methionine (Figure 1C). This prediction held for the *A. aegypti* gene, even when initiation occurred at the second methionine, though it was not as strong for *A. albopictus* and *C. quinquefasciatus* orthologs. However, using the online prediction tool ATGpr [27], the first methionine was predicted to be in a better context than the second. Furthermore, amino acid conservation between the three culicine mosquitoes extends upstream of the second methionine; evidence that it is likely the first start codon is preferred. Ultimately, experimental evidence will be necessary to confirm these predictions and the potential significance of a single membrane-bound CTLDcp.

Six proteins were classified as CTLD-X: all share the same domain structure with 1:1 counterparts, as described in lepidoptera [8,9]. Three of the CTLD-X proteins contain multiple copies of the CCP/sushi domain that is associated with the complement arm of the immune system, and were previously identified as restriction factors for DENV or YFV [28]. Five of the six CTLD-X proteins are predicted to be membrane-associated with all identified domains present in extracellular topological space with unstructured cytoplasmic tails. The sixth protein, containing multiple immunoglobulin repeats, may or may not be membrane associated as well, as a C-terminal transmembrane domain was predicted with only ~40% confidence. Finally, we placed proteins CLSP1 and CLSP2 [7] in a new group, CTLD-SP. A third protein, which is termed here CLSP3, was identified, which had a similar domain structure of N-terminal serine protease and C-terminal CTLD. We considered categorizing these as CTLD-X, as they are CTLDcps with additional known domains, but did not for several reasons. Notably, the CTLD-X proteins that are described in lepidopterans (and here in Aedes) have a complex structure with multiple independent domains; no single moniker could succinctly describe them. In addition, the CTLD-X proteins are all highly conserved. In contrast, the domain structure of CTLD-SP proteins is simple and they are so far only found in culicine mosquitoes, making the use of a unique designation appropriate and informative.

We compared the length distribution of CTLDcps to those that are currently annotated in three other mosquito genome assemblies: 40 CTLDcps in *A. albopictus* [29], 64 in *C. quinquefasciatus* [30], and 38 in *A. gambiae* [31]. For *A. aegypti*, CTLD-S members were found to be extremely uniform in size, with a median length of 162 amino acids (a.a.; Figure 1D). Thirty-one of 34 were found in a narrow range from 147 to 175 a.a., while two were unusually short (114 and 116 a.a.) due to internal deletions in the CTLD. CTLD-E members ranged from 217 to 738 a.a., with CTLD-SP members clustering between 429–450 a.a. As would be expected based on their complex domain structure, CTLD-X members were the largest, ranging from 1052 to 3586 a.a. These size ranges may serve as useful proxies, particularly as new mosquito genomes are sequenced and annotated. When compared to the other three mosquitoes, a similar clustering pattern was observed, although the clustering around the CTLD-S size range was not as tight. It is likely, at least for the other two culicine mosquitoes, that this represents (at least in part) the limitations of the current assemblies and automated gene prediction pipelines.

### 3.2. Developmental Expression Profile of A. aegypti CTLDcps

**Figure 2:**
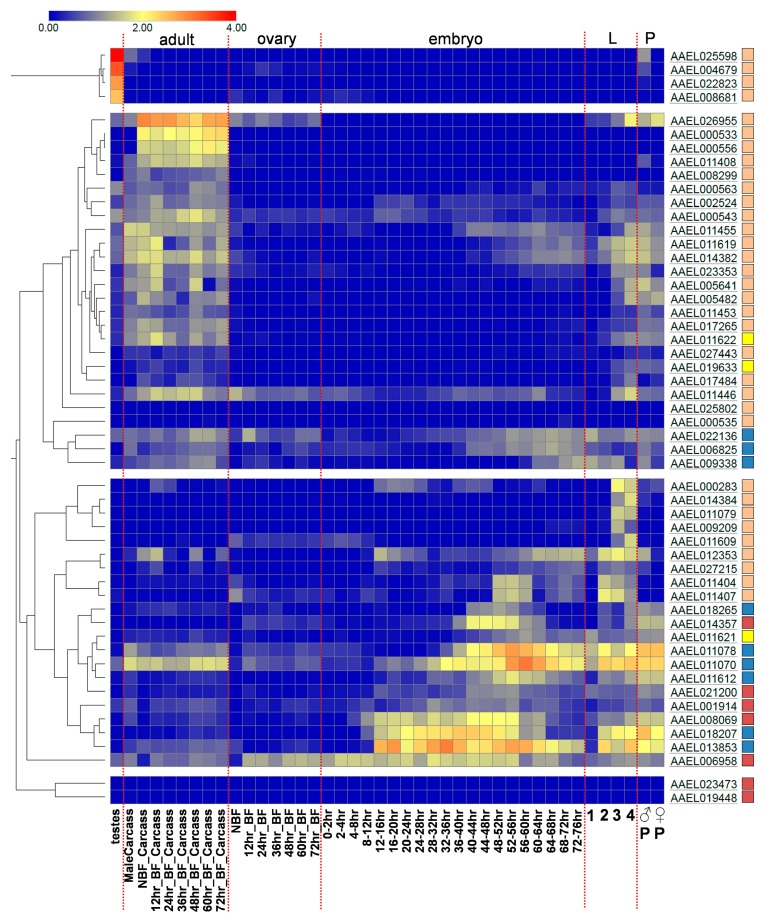
Developmental expression of *A. aegypti* mRNA transcripts encoding CTLDcps. Color indicates the log10 FPKM expression value. No scaling is applied to rows; clustering was performed using Morpheus (https://software.broadinstitute.org/morpheus) with one minus pearson correlation and average linkage. CTLD-S (■), -SP (■), -E (■), and -X (■) groups are indicated. NBF, non-bloodfed; BF, bloodfed; L, larvae; P, pupae.

To characterize CTLDcps based on the expression profile of their encoding mRNA, we obtained normalized expression values during a series of developmental stages [18] that had be re-mapped to the AaegL5 assembly (Figure 2). Notably, most members of groups CTLD-X and CTLD-E clustered together with similar expression patterns beginning in 12–16 h embryos and continuing through pupal development, with reduced expression in adult stages. Conversely, members of the CTLD-S and CTLD-SP groups largely clustered together with expression generally beginning around the third instar stage and ending in the fourth instar, or beginning in the third instar and continuing into the adult, with a few notable exceptions. Two CTLD-S genes (AAEL000533 and AAEL000556) were observed to be specifically expressed in adult females. Indeed, the corresponding proteins were previously identified as specific to the female salivary glands [26] and were recovered with other salivary components in the guts of newly fed females [32]. An additional four CTLD-S genes (AAEL025598, AAEL008681, AAEL004679, and AAEL022823) were all highly expressed, specifically in the testes. Finally, we note that AAEL026955, a new gene not previously included in any reverse genetic analysis of immunity or virus infection, was by far the most abundantly transcribed CTLDcg in adult females.

### 3.3. Phylogeny of Mosquito CTLDs

**Figure 3:**
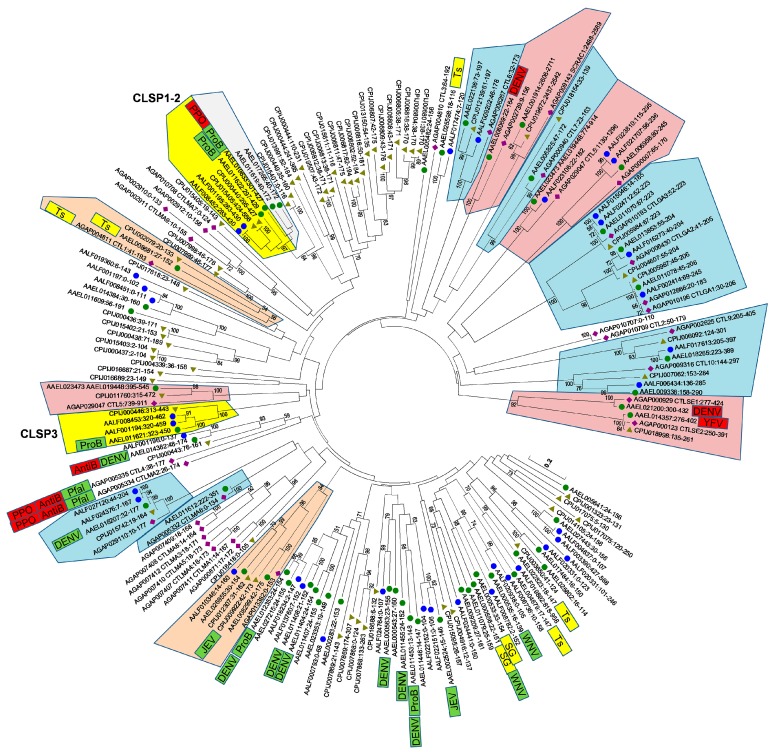
Neighbor-joining tree based on alignment of 183 CTLDs extracted from *A. aegypti* (●), *A. albopictus* (●), *C. quinquefasciatus* (▲), and *A. gambiae* (♦) CTLD containing genes. Ambiguous positions were removed for each sequence pair; there were a total of 235 positions in the final dataset; branch support of more than 50% (1000 replicates) is indicated. Conserved clades containing CTLD-X (red) and CTLD-E (blue) members are indicated. Other highlighted clusters contain CTLD-SP (yellow) and CTLD-S proteins with both *A. gambiae* and *A. aegypti* orthologs with branch support over 50% (orange). Individual CTLDcps previously implicated as host (green) or resistance (red) factors for dengue virus (DENV), Japanese encephalitis virus (JEV), West Nile encephalitis virus (WNV), *P. falciparum* (Pfal) bacteria (ProB/AntiB), or the prophenol oxidase response (PPO) are indicated, as are genes specifically expressed in the salivary glands (SG) and testes (Ts).

Waterhouse et al. [5] noted nine orthologous trios and a series of insect-specific expansions when comparing the CTLDcps of *A. aegypti*, *A. gambiae,* and *D. melanogaster*. With the identification of 13 additional *A. aegypti* CTLDs, as well as the availability of two additional culicine mosquito genomes, we anticipated that the evolutionary history of CTLDcps may be better resolved. After the extraction of all CTLDs (*n* = 189) from each of the four mosquito genomes, we performed a multiple sequence alignment and constructed a Neighbor-joining tree to visualize the relationships between them (Figure 3). Several features are readily apparent. Sixteen groups (corresponding to 15 genes, since the CTLD-X5 ortholog contains two CTLDs) contained at least one *A. gambiae* and one *A. aegypti* member with >90% branch support). Most of these groups had *C. quinquefasciatus* and *A. albopictus* members as well, although missing/duplicated genes here may reflect the quality of the respective genome assemblies/annotations. Notably, these high confidence orthologous groups comprise all CTLD-X (*n* = 6) and CTLD-E (*n* = 9) clusters, and none of the CTLD-S or CTLD-SP genes. Just two CTLD-S clades include both an *A. gambiae* and an *A. aegypti* gene with substantial branch support. In one case, two *A. aegypti* CTLD-S proteins (AAEL008299 and AAEL026955) group with an *A. gambiae* counterpart (AGAP013382) at 84% branch support, along with two *C. quinquefasciatus* and one *A. albopictus* protein. The other orthologous group contained AAEL008681 (one of the four testes-specific CTLDcps) and groups with one protein from each of the other three mosquitoes at 56% branch support. Confidence in this relationship is boosted by the fact that the *A. gambiae* gene in this clade is also known to be expressed specifically in the testes [33], suggesting that these genes may in fact be true orthologs, albeit rapidly evolving ones. Finally, *A. gambiae* CTLD-S genes CTL4 and CTLMA2, whose protein products are known to heterodimerize as a part of the humoral immune response against gram negative bacteria, while also inhibiting melanization to the benefit of malaria parasites [34,35], group most closely with CTLD-S protein AAEL014382 (CTLMA14; mosGCTL-24), albeit with weak support (23%). Interestingly, AAEL014382 (mosGCTL-24) was found to be important for the antibacterial immune response in *A. aegypti* as well [6], offering some support for a role as functional orthologs. Just as the loss of AgCTL4/AgCTLMA2 resulted in a decrease in Plasmodium parasites in *A. gambiae* [34], knockdown of AAEL014382 resulted in a decrease in DENV replication in *A. aegypti* [3]. We caution that, although we find strong support amongst individual clades throughout the tree, the deeper branches that establish how different clades are related to each other are all poorly supported and they should be interpreted with caution.

**Figure 4:**
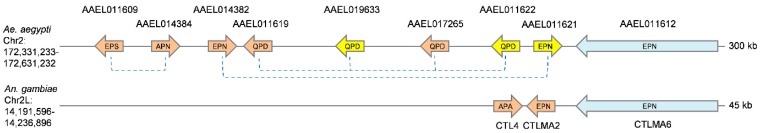
Expansion of CTLD-S and CTLD-SP genes in *A. aegypti*. Syntenic regions of the *A. aegypti* and *A. gambiae* genomes containing related clusters of CTLDcps. Genes encoding CTLD-S (orange), CTLD-SP (yellow), and CTLD-E (blue) are indicated (not to scale). Dotted lines indicated *A. aegypti* CTLDs that clustered together in Figure 3. Three letter codes indicate the residues present in the putative sugar-binding region of each CTLD.

CTLD-SP family members were identified in both *C. quinquefasciatus* and *A. albopictus*, suggesting that the fusion events that created these novel genes occurred before these species diverged from each other. Interestingly, the CTLDs of CLSP1 and CLSP2 were found to be distinct from that of the third gene, CLSP3. In fact, while both CLSP1/CLSP2 have the conserved QPD motif that is typical of galactose-binding, CLSP3 bears an EPN motif associated with mannose-binding. Thus, we considered the possibility that over the evolutionary history of culicine mosquitoes, a serine protease fused with a CTLD on not one, but two separate occasions. A prediction of such an unlikely scenario would be that the serine protease domains of CLSP3 and CLSP1/2 would each have distinct best matches to potentially ancestral serine protease genes. We performed a blastp-based search using the first 270 a.a. of each CTLD-SP gene (consisting of the signal peptide and serine protease domain only) to identify the closest matches from the AaegL5.1 geneset. Surprisingly, the best match for both CLSP1 and CLSP2 was the serine protease domain of CLSP3 (Table 1). The reverse was not true, as six other serine proteases were better scoring matches than CLSP1/2 (Table 1). We hypothesize that CLSP3 represents the initial gene fusion event between a serine protease and a CTLD-S. Sometime after, but still before the divergence of Culex and Aedes, this fusion gene was duplicated; subsequently, the EPN-based CTLD was replaced by an alternative QPD version, perhaps by ectopic homology-based repair. Such events can be facilitated when related genes are present as part of a larger gene cluster. To investigate this possibility, we analyzed the genome region surrounding the *A. aegypti* CTLD-SP genes (Figure 4). Within a 300 kb region, we identified a cluster of nine genes encoding CTLDcps (1 CTLD-E, 5 CTLD-S, and all three CTLD-SP genes), with no intervening unrelated genes. This was found to be the largest CTLD cluster in the *A. aegypti* genome (see Table S1; another cluster on chromosome 3 contains six genes encoding CTLDcps, with six additional mini-clusters containing 2–3 genes). CTLD-S genes present in the largest cluster were of the EPN (AAEL013482) and QPD (AAEL017265 and AAEL011619) type, providing the necessary raw material for an evolutionary switch in regards to CTLD-SP genes. This region also appear to be syntenic with the *A. gambiae* CTLMA2/CTL4 locus, the known antibacterial factors that inhibit the humoral melanization response, and thus these gene expansions have important implications for immune diversity in Aedes mosquitoes. Several CTLDcps in this cluster have been found to be induced by bacterial challenge, with regulation being controlled by both the Toll and Imd pathways [6,7,22]. Ultimately, additional genome sequences of more distant culicine mosquitoes will be necessary to establish how these genes duplicated and fused to their current state.

### 3.4. Virus-Induced Expression of Transcripts Encoding CTLDcps

**Figure 5:**
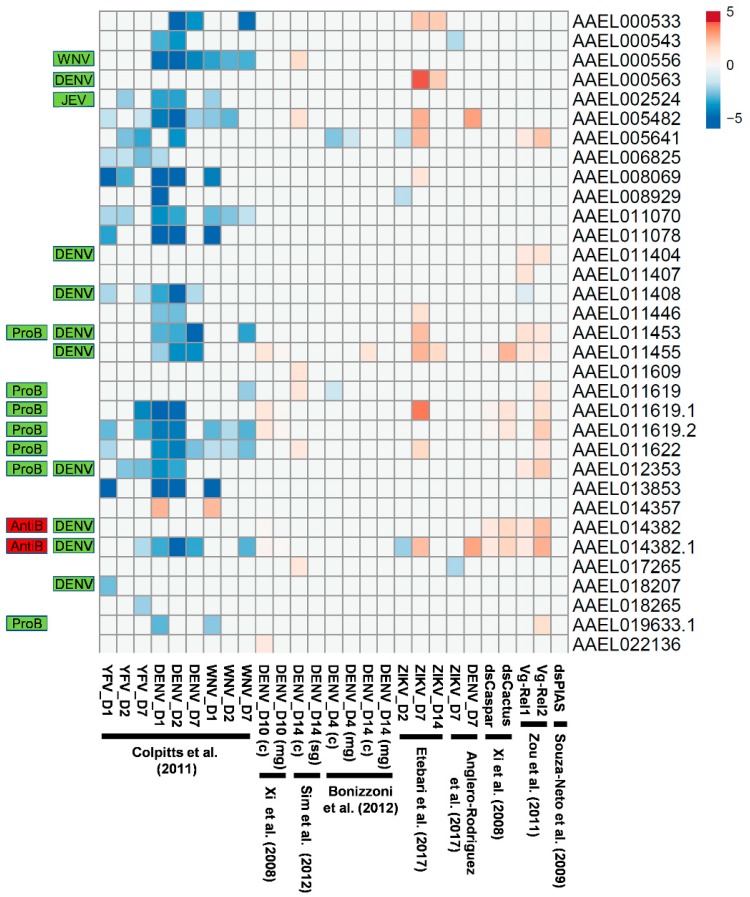
Differentially-regulated CTLDcp-encoding transcripts after virus challenge or immune activation. Heat map of log2 values corresponding to the change in gene expression after virus infection [20,21,23,24,25,37], dsRNA treatment [21,36], or bloodmeal-induced activation of Rel1/Rel2 [22]. Only those genes described as being significantly different in the initial publications are included. Transcripts that are associated with CTLDcps identified as virus host factors (DENV, JEV, WNV) [1,2,3] or probiotic (ProB)/antibiotic factors (AntiB) [6] are indicated.

Transcripts encoding CTLDcps have been shown to be induced by bacterial [6,7] as well as virus infection [1,2,3]. In particular, the upregulation of CTLD-S genes that also serve as viral host factors may play a role in exacerbating the replication of arboviruses in mosquitoes, rather than in controlling them. To determine whether this is a general phenomenon, we sought to compare these results with published high-throughput expression experiments where gene expression changes were monitored in an unbiased manner through microarray or Illumina-based sequencing [20,21,22,23,24,25,36,37]. In one study, twenty-four transcripts encoding CTLDcps were found to have significantly decreased expression following infection with YFV, DENV, or WNV (1, 2, or 7 days after infection), with just one increasing in expression (Figure 5; [23]). In contrast, Bonizzoni et al. [37] observed a decrease in the expression of just two CTLDcp-encoding transcripts at four days after infection with DENV, while Anglero-Rodriguez [24] observed the upregulation of two CTLDcp-encoding transcripts at seven days after infection with DENV. This was similar to data reported by Xi et al. [21] and Sim et al. [20], where 4–6 CTLDcp-encoding transcripts were found to be upregulated late in the infection process (10–14 days after DENV infection). Etebari et al. [25] observed a similar pattern following the infection of *A. aegypti* with ZIKV, where several CTLDcp-encoding transcripts were downregulated early in the infection (day 2), followed by the exclusive upregulation of a subset at days 7 and 14. Of the nine CTLDcps found to be host factors for DENV [3], only one (AAEL011455) had an associated transcript upregulated after DENV infection in any of these studies. However, the transcript encoding the primary DENV host factor mosGCTL-3 (AAEL000563) was found to be strongly upregulated in ZIKV-infected mosquitoes at both 7 and 14 days after virus challenge (Figure 5; [25]). Transcripts associated with six of the nine DENV-associated host factors [3] and all microbiome-regulating CTLDcps [6] were also regulated by the Toll and/or Imd pathways. Given the known association between the Toll pathway controlling DENV infection [21], as well as the microbiome influencing DENV infection [21,38], it is no understatement to say that CTLDcps appear to have a complex role in influencing arbovirus replication.

## 4. Discussion

In vertebrates, proteins containing a C-type lectin fold are important both in the antiviral response to arboviruses, such as DENV [39], but also as important host factors that bind viral particles and promote their entry into and the subsequent infection of host cells [40,41]. Since the initial discovery that mosquito CTLDcps also bind and promote the entry of WNV into mosquito cells [1], CTLDcps have also been shown to be important for DENV and JEV infection of mosquitoes [2,3]. All of these experiments have demonstrated that the depletion of CTLDcps reduces viral RNA levels, while transient overexpression increases viral RNA levels. Most importantly, antibodies that bind CTLDcps can reduce infection levels in mosquitoes [1,2,3]. Interestingly, there is no overlap to date between the CTLDcps shown to interact with each of these viruses, though 11 out of 12 were classified here as members of group CTLD-S. With a more complete annotation of CTLDcps now available, we note that only 16/34 (47%) CTLD-S proteins were included in the screen for an effect on WNV replication [1], while 25/34 (74%) were tested for an effect on DENV [3]. Thus, it is possible that additional CTLDcps that promote the replication of these viruses remain to be discovered. While the expression of many CTLDcps was reported to increase following JEV infection, just two of these were experimentally analyzed in *A. aegypti*: AAEL002524 (mosGCTL-7) was found to bind directly to JEV and it was important for wild-type replication levels and AAEL008299, whose loss of function had no effect on JEV replication [2]. Interestingly, this same group identified a *C. quinquefasciatus* homolog (CPIJ009922) that also was found to be important for JEV infection [2]. However, our comparison of all CTLDcps from the four mosquitoes showed that CPIJ009922 is in fact a 1:1 ortholog of AAEL008299, and it is only distantly related to AAEL002524. This finding, along with the general dispersal of virus host factors amongst CTLD-S gene clades suggests that the ability to bind (and hence, assist) different flaviviruses can be gained and lost relatively easily over evolutionary time. It seems likely that CTLDcps will be shown to interact with other medically important flaviviruses, such as YFV and ZIKV, though experimental evidence is currently lacking. Just as interesting would be determining if CTLDcps are also important in the infection and maintenance of insect-specific flaviviruses, where competition for such host factors may restrict the replication of arboviruses [42].

In addition to serving as viral host factors, mosquito CTLDcps have been shown to have important roles as negative regulators of the mosquito melanization response [7,34,43], the Toll immune pathway [43], and the antimicrobial peptide response [6]. Depletion of CTLMA2/CTL4 in *A. gambiae* resulted in an increase in melanization to the detriment of invading malaria parasites [34]. Similarly, depletion of CLSP2 in *A. aegypti* increases the melanization response to both malaria parasites [7] and fungi [43], in the latter case, also reducing pathology that is associated with fungal infection. While not direct orthologs, we found that these genes occupy syntenic gene clusters that were independently duplicated in both Anopheles (once) and Aedes (multiple times), potentially as a result of natural selection acting on dampening the activity of these immune responses. Interestingly, CLSP2 was observed to be post-translationally cleaved upon fungal infection into its constituent protease and CTL domains, with the freed CTLD able to bind molecules on the fungal surface in a calcium-dependent manner [43]. Transcriptional profiling of mosquitoes following the depletion of CLSP2 demonstrated a dramatic upregulation of Toll-pathway pattern recognition molecules and effectors, suggesting that these are normally suppressed by CLSP2 in a manner that remains unknown. The role of CLSP1 remains unclear, as well as that of the newly described gene CLSP3, both of which we found located in the same Imd/Toll-regulated gene cluster. Finally, CTLDcps have been shown to bind directly to bacterial surfaces and prevent the binding and killing effects of anti-microbial peptides [6]. This protective effect on the gut microbiome was found to be mediated by the Imd pathway [6], as is consistent with previously published microarray experiments that observed the upregulation of transcripts of the same CTLDcps after Imd activation (Figure 5; [21,22]).

Two CTLD-S proteins (AAEL0011453 and AAEL012353) were identified as being important for both protecting the gut microbiome [6] and for facilitating DENV infection [3]. This raises the possibility that there could be competition in the mosquito gut for binding to CTLDs between any resident bacteria and invading flaviviruses. Indeed, higher levels of bacterial colonization have been associated with reduced susceptibility to DENV infection, while lower bacterial counts resulted in an increase in infection [21]. This was hypothesized to be mediated through an increase/decrease in immune activation, with antimicrobial peptides being activated by the microbiome acting against the invading viruses [21]. The composition of the microbiome can also influence DENV infection rates [38], and while differential immune stimulation may play a role in this as well, variation in CTLD binding to different resident bacterial surfaces (particularly amongst those that also can interact with arboviruses), may also contribute to variance in infection rates and virus replication success. Conversely, reported dsRNA or antibody treatments that deplete or sequester one or more CTLDcps may impact microbiome abundance or composition, potentially resulting in additional indirect effects on arbovirus replication. Differences in microbiome composition may have contributed to the large variation that we observed in transcript modulation following flavivirus infection across different published experiments, where transcripts encoding the same CTLDcps were found to decrease, increase, or remain unchanged across experiments. While this may partially reflect the sensitivity of the assays used (microarray/RNAseq), more clarity into how different flaviviruses might trigger transcriptional increases of host factor CTLDcps is certainly needed. We also note that only two out of four DENV-associated CTLDcps were identified, both in an initial screen [1] and in a larger subsequent screen [3]. Differences in microbiome or baseline immune status between these two trials may have contributed to masking the effects of these proteins, indicating that even more CTLDcps that facilitate flavivirus infection may yet be described.

In summary, we have expanded the catalog of *A. aegypti* CTLDcps from 39 to 52 and attempted to group these based on protein domain architecture. Phylogenetic comparisons with other mosquitoes combined with data mining of published expression profile studies support the classification of CTLDcps into functionally distinct subgroups. We found that CTLD-X and CTLD-E members are evolutionarily conserved and largely developmentally expressed, while CTLD-S and CTLD-SP members are largely expressed from late larval stages through the adult, with some acquiring expression patterns that are specific to reproductive tissues (testes) or salivary glands. At least 12 CTLDcps were found to be regulated by the Toll/Imd immune pathways, and all were CTLD-S and CTLD-SP genes. Likewise, 11/12 flavivirus-associated host factors and all microbiome-modulating CTLDcps were classified as CTLD-S. Additional genome sequencing efforts targeted to other culicine mosquitoes will help to clarify the evolutionary relationships between CTLD-S members. Such efforts may also help identify those CTLD-S genes under the strongest selection pressure, which in turn may point to specific pathogens/microbes responsible for shaping the evolution of these important disease vectors.

## Figures and Tables

**Table 1:** Relationship between serine protease domains of CTLD-SP genes and potential ancestral serine proteases in the *A. aegypti* genome.

**CLSP-1 (Signal P and SP Domain Only)**					
**CTLD-SP**	**Gene ID**	**Length**	**Evalue**	**Score**	**% Identity**
CLSP-1	AAEL011622-PA	270	0	1442	100%
CLSP-2	AAEL019633-PA	270	5.00E-180	1305	90%
CLSP-3	AAEL011621-PA	253	9.00E-64	533	38.90%
	AAEL019963-PA	262	2.00E-55	471	34.20%
	AAEL005748-PA	244	1.00E-52	454	37.60%
	AAEL018109-PB	245	8.00E-51	450	37.90%
	AAEL002276-PA	241	7.00E-51	436	36.10%
	AAEL001077-PA	245	5.00E-48	432	38.70%
	AAEL010773-PA	246	7.00E-48	431	36.40%
	AAEL001084-PA	245	2.00E-47	427	38.30%
**CLSP-2 (Signal P and SP Domain Only)**					
CLSP-2	AAEL019633-PA	270	0	1441	100%
CLSP-1	AAEL011622-PA	270	4.00E-180	1306	90%
CLSP-3	AAEL011621-PA	262	2.00E-65	544	37.70%
	AAEL019963-PA	262	2.00E-52	451	34.60%
	AAEL018109-PB	245	8.00E-50	443	37.90%
	AAEL010773-PA	246	1.00E-48	436	35.50%
	AAEL001084-PA	245	1.00E-47	429	37.90%
	AAEL005748-PA	244	1.00E-48	426	35.90%
	AAEL001077-PA	245	9.00E-46	415	38.20%
	AAEL006253-PC	243	2.00E-45	412	35.10%
**CLSP-3 (Signal P and SP Domain Only)**					
CLSP-3	AAEL011621-PA	270	0	1435	100%
	AAEL011375-PB	234	8.00E-67	559	44.90%
	AAEL010773-PA	233	4.00E-62	534	43.20%
	AAEL018109-PB	259	2.00E-58	505	37.30%
	AAEL002880-PA	266	1.00E-57	499	37.50%
	AAEL002254-PA	263	5.00E-57	498	39.10%
	AAEL019963-PA	237	4.00E-59	497	39.20%
CLSP-2	AAEL019633-PA	257	1.00E-58	497	36.60%
	AAEL002276-PA	242	1.00E-59	496	41.10%
CLSP-1	AAEL011622-PA	238	1.00E-57	490	38.30%

## Supplementary Materials

Supplementary materials can be found at http://www.mdpi.com/1999-4915/10/7/367/s1.
